# Vasopressin antagonist-like effect of acetazolamide in a heart failure patient: a case report

**DOI:** 10.1093/ehjcr/yty076

**Published:** 2018-07-02

**Authors:** Hajime Kataoka

**Affiliations:** Division of Internal Medicine, Nishida Hospital, Tsuruoka-Nishi-Machi 2-266, Saiki-City, Oita, Japan

**Keywords:** Case report, Heart failure, Diuretics, Acetazolamide, Hyponatraemia, Vasopressin antagonist

## Abstract

**Background:**

Hyponatraemia is easily corrected by treatment with an oral vasopressin antagonist, but these medications are costly and their use at outpatient clinics is restricted by government-managed insurance in Japan. Acetazolamide could be an alternative diuretic to a vasopressin antagonist.

**Case Summary:**

An 83-year-old dyspnoeic male patient was emergently admitted to the hospital due to decompensated heart failure (HF), hypotension, and hyperkalaemia-associated sinus arrest with a junctional escape rhythm. Urgent treatment with a noradrenaline drip infusion and a beta stimulant adhesive skin patch promptly restored sinus rhythm with conducted normal QRS complex, which resolved the hypotension. Blood tests on admission revealed moderately elevated b-type natriuretic peptide (BNP, 576 pg/mL), hyponatraemia (128 mEq/L), hypochloraemia (95 mEq/L), hyperkalaemia (5.7 mEq/L), and preserved renal function (creatinine, 1.0 mg/dL) under no cardiovascular medications. Immediately after admission, low-dose oral acetazolamide (500 mg/day) and polystyrene sulfonate-Ca jelly (Argamate, 25 g/day for 3 days) were prescribed to correct the decompensated HF status and electrolyte disturbance. Three days later, both the serum sodium and chloride concentrations had recovered to normal levels (136 mEq/L and 104 mEq/L, respectively), and the serum potassium concentration had decreased to 4.5 mEq/L. Two weeks later, the patient’s HF status became stable and the serum BNP concentration returned to normal (55 pg/mL).

**Discussion:**

The present case indicates that the classic diuretic of acetazolamide would have a vasopressin blockade-like effect and could be an alternative diuretic to vasopressin antagonists for some proportion of HF patients with hyponatraemia.

**Learning points**

- The patient reported here suggests that acetazolamide could be an alternative diuretic to vasopressin antagonists for some proportion of heart failure (HF) patients with hyponatraemia. This observation is supported by literature describing that the serum sodium concentration is preserved or enhanced in 70% of refractory HF patients undergoing acetazolamide treatment.
- Large randomized studies are required to re-evaluate the efficiency of this forgotten, but useful agent for the treatment of HF and its potential for correcting hyponatraemia.

## Introduction

Hyponatraemia in acutely decompensated heart failure (HF) is easily corrected by vasopressin receptor antagonists,^1–3^ but these medications are costly and their use in outpatient clinics is restricted in Japan by government-managed insurance. According to the ‘chloride theory’ for HF pathophysiology,^4–6^ chloride manipulation, including the use of acetazolamide (Diamox),^7–9^ may be an essential therapeutic target in HF treatment. Previous reports revealed that chloride is a crucial electrolyte for reabsorption of filtered sodium in the renal tubules to the extracellular space of the body.^10–12^ Acetazolamide could become an alternative diuretic to an oral vasopressin antagonist for correcting hyponatraemia due to more aquaresis than natriuresis because; (i) this diuretic agent enhances the reabsorption of chloride,^5–7^ and thus sodium is expected to be reabsorbed concurrently in the cortical collecting tubules^10–12^ and (ii) might have an ability of increasing the free water clearance in the urinary tubules.^13^

## Timeline

**Table:** 

Day	Events
1	The patient was emergently admitted to the hospital due to acutely decompensated heart failure (HF) (NYHA-IV) with hypotension (60/42 mmHg) and bradycardic junctional rhythm (30 b.p.m.)
	Cardiac transthoracic ultrasound revealed moderate degree of aortic regurgitation (III/IV), but a preserved left ventricular ejection fraction (60%)
	Blood tests revealed moderately elevated b-type natriuretic peptide (BNP 576 pg/mL), hyponatraemia (128 mEq/L), and hypochloraemia (95 mEq/L)
	Urgent noradrenaline drip infusion (2–3 μg/kg/h) promptly restored sinus rhythm (70 b.p.m.) and led to recovery from hypotension
	Low-dose oral acetazolamide (500 mg/day) was prescribed to correct the decompensated HF status and electrolyte disturbance
4	Both the serum sodium and serum chloride concentrations recovered to normal (136 mEq/L and 104 mEq/L, respectively)
14	The patient’s HF status became stable, and the serum BNP concentration returned to normal (55 pg/mL), but the serum sodium was slightly reduced (133 mEq/L)
21	The patient was discharged from the hospital in an acceptable HF status
21 + 2 months	The HF status remained to be stable (serum BNP level of 65 pg/mL) and both the serum sodium and chloride concentrations were normal (139 mEq/L and 108 mEq/L, respectively)

## Case presentation

An 83-year-old male patient was emergently admitted to the hospital with the chief complaint of progressive dyspnoea over several days due to acutely decompensated HF (NYHA-IV) with hypotension (60/42 mmHg), bradycardia, and an irregular pulse (30 b.p.m.). The patient was uneventful for recent several years, and was not on any regular medication, including cardiovascular drugs, before the present admission. *Table 1* shows the clinical course of his HF-related tests, peripheral blood and urinary tests, and medications given to treat the decongestion and electrolyte disturbance. Physical examination on admission revealed jugular venous distension, systemic oedema, bilateral basal pulmonary rales, distant heart sound, and peripheral coldness. A 12-lead electrocardiogram revealed sinus arrest with a junctional escape rhythm and an irregular heart rate of 30 b.p.m. A chest X-ray revealed mild cardiomegaly (cardiothoracic ratio 55%) and prominent vasculature in the upper lung fields. Transthoracic cardiac ultrasound revealed a moderate degree of aortic regurgitation (III/IV), but the left ventricular ejection fraction (60%) was preserved, and its diastolic volume was almost within the normal range (143 cc). Thoracic and abdominal ultrasound showed massive bilateral pleural effusion and an expanded inferior vena cava with minimal respiratory change.

**Table 1:** Changes in serum and urinary electrolytes before and after the administration of Diamox

	Admission to the hospital (24 October 2017)						
	Before Diamox treatment		After Diamox treatment				
	Day 1		Day 4		Day 8		Day 14
	At admission	8 h later					
A. Heart failure-related test							
Urine volume (mL/d)		1000–2100–1400–1000			1400		—
Blood pressure (mmHg)	60/42	117/56	118/60/		106/56		96/59
Heart rate (b.p.m.)	30	70	83		78		68
Electrocardiogram	Sinus arrest/escape rhythm	NSR/conducted narrow QRS	—		—		NSR/conducted narrow QRS
Body fluid retention (physical and ultrasonographic exam)	Systemic oedema and pleural effusion		—		—		No
B-type natriuretic peptide (pg/mL)	576		—		—		55
Renin activity (ng/mL/h)	0.9		—		—		2.7
Anti-diuretic hormone (pg/mL)	8.6		—		—		1
B. Peripheral blood test							
Haemoglobin (g/dL)	11.5		10.9		10.9		10.2
Haematocrit (%)	34.4		35.9		35		32.5
MCV (fL)	97		106		104		102
Serum electrolytes							
Sodium (mEq/L)	128		136		135		133
Potassium (mEq/L)	5.7		4.5		4.1		4.2
Chloride (mEq/L)	95		104		103		103
Blood urea nitrogen (mg/dL)	33		23		23		20
Serum creatinine (mg/dL)	1		0.77		0.6		0.65
C. Urinary test (spot urine)							
Urinary electrolytes							
Sodium (mEq/L)	104		—		—		113
Potassium (mEq/L)	40		—		—		34
Chloride (mEq/L)	100		—		—		98
Urinary creatinine (mg/dL)	44		—		—		56
Urinary electrolytes corrected by urinary creatinine							
Sodium/Cr (10 mEq/g·Cr)	2.36		—		—		2.02
Potassium/Cr (10 mEq/g·Cr)	0.91		—		—		0.61
Chloride/Cr (10 mEq/g·Cr)	2.27		—		—		1.75
D. Treatment							
Noradrenaline	2–3 μg/kg/h						
Beta stimulant skin patch	2 mg/d (Tulobuterol)						
Argamate 20% jelly		25 g/d					
Carbonic anhydrase inhibitor		500 mg/d (Diamox)					

Cr, Creatinine; d, day; h, hour; MCV, mean corporeal red cell volume; NSR, normal sinus rhythm.

Urgent initiation of a noradrenaline drip infusion (2–3 μg/kg/h) and beta stimulant adhesive skin patch (Tulobuterol 2 mg/day) promptly restored the sinus rhythm (70 b.p.m.) and the normality of conduction and the QRS complex on electrocardiography, resulting in the recovery from hypotension with a systemic blood pressure of 117/56 mmHg. Blood tests on admission revealed moderately elevated b-type natriuretic peptide (BNP 576 pg/mL; normal range <18.4 pg/mL), hyponatraemia (128 mEq/L; normal range 135–147 mEq/L), hypochloraemia (95 mEq/L; normal range 98–108 mEq/L), hyperkalaemia (5.7 mEq/L, normal range 3.6–5.0 mEq/L), and preserved renal function (creatinine 1.0 mg/dL; normal range 0.61–1.04 mg/dL) under no particular cardiovascular medications. Immediately after resolving the bradycardia and hypotension, low-dose oral acetazolamide (500 mg/day) and 20% polystyrene sulfate-Ca jelly (Argamate 25 g/day for 3 days) were prescribed to correct the decompensated HF status and electrolyte disturbance. Three days later, both the serum sodium and chloride concentrations had recovered to normal levels (136 mEq/L and 104 mEq/L, respectively), and the serum potassium concentration had decreased to 4.5 mEq/L.

Two weeks later, the patient’s body fluid retention was ameliorated and the serum BNP concentration was near normal (55 pg/mL). Serum chloride and potassium concentrations remained in the normal range, but the serum sodium concentration was slightly reduced (133 mEq/L) compared to that at Day 4 of the hospital admission, which could be managed by changing the diuretic prescription and/or dietary salt adjustment in accordance with the ‘chloride theory’ for HF pathophysiology.^6^ Three weeks later, the patient was discharged in an acceptable HF status. Two months later after discharge under the same medication, the patient’s HF status remained stable (serum BNP level of 65 pg/mL) and both the serum sodium and chloride concentrations were normal (139 mEq/L and 108 mEq/L, respectively).

## Discussion

The present case indicates that the classical drug acetazolamide not only has a potent chloride-regaining diuretic effect but also has a vasopressin blockade-like effect to enhance the serum sodium concentration presumably through the mechanism of more aquaresis than natriuresis by this diuretic agent. Hyponatraemia was promptly corrected (within 4 days in this case) after acetazolamide monotherapy.

Electrolyte abnormalities may be multifactorial and interrelated, resulting from neurohormonal activation, renal dysfunction, medications, and dietary intake.^14^ In the present study, hyponatraemia and hypochloraemia on admission were associated with enhanced activation of anti-diuretic hormone (8.6 pg/mL; normal range <2.8 pg/mL) and normal renin activity (0.9 ng/mL/h; normal range 0.3–2.9 ng/mL/h). Under the situation of both hyponatraemia and enhanced activation of anti-diuretic hormone, vasopressin receptor blockade would be the primary choice of diuretics.^1^^,^^2^ Acetazolamide was selected for this patient, however, because recent studies have suggested that manipulating the serum chloride concentration is important for improving the haemodynamic and neurohormonal status in HF pathophysiology.^3^^,^^6^ As a consequence, this diuretic strategy using low-dose oral acetazolamide corrected both the hyponatraemia and hypochloraemia in parallel with improvement of the worsening of HF status. Additionally, hyperkalaemia was simultaneously corrected by this agent due to its potential to reduce serum potassium concentration by secreting this electrolyte into the urinary tubules.^7–9^ At Day 14 of hospitalization and after acetazolamide treatment (*Table 1*), the plasma renin activity was slightly increased, but it remained normal (2.7 ng/mL/h), and the anti-diuretic hormone level was decreased to normal level (1 pg/mL).

**Figure 1:**
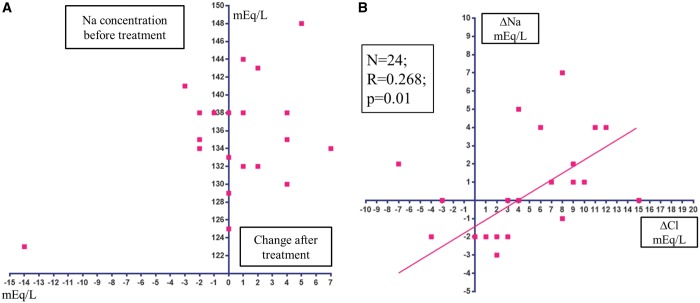
(*A*) Scatterplot of changes in the serum sodium concentration after treatment with acetazolamide. (*B*) Correlation between changes in the serum chloride concentration and serum sodium concentration after treatment with acetazolamide. Data obtained in the study by Relman *et al.*^7^

The potential of acetazolamide to correct hyponatraemia is expected based on previous studies. The carbonic anhydrase inhibitor acetazolamide has a unique but critical diuretic action, a ‘non-reabsorbable anion-like effect’, that results in the excretion of bicarbonate (HCO_3_^−^) into the urinary tubules with interchangeable absorption of filtered chloride into the blood, and concurrent excretion of potassium into the urine.^7–9^ An earlier study by Relman *et al.*^7^ examined the acute effects (3–12 days) of acetazolamide on serum solutes in 26 patients with severe HF (male 73%; age 56.7 ± 12.4 years) and found that the serum sodium concentration was unchanged or increased in 73% of 24 evaluations from the 23 HF patients (*Table 2* and *Figure 1A*). Furthermore, the increased serum sodium concentration correlated positively with the increased serum chloride concentration (*Figure 1B*), suggesting that the reabsorption of chloride might be enhanced concurrently with the reabsorption of sodium in the urinary tubules to the blood stream.^8^^,^^10–12^ More importantly, acetazolamide might have aquaretic effects,^13^ presumably through the ‘non-reabsorbable anion-like effect’,^8^ that might enhance serum sodium concentration. It is required furthermore to examine the exact mechanism(s) for enhancing serum sodium concentration by acetazolamide.

**Table 2:** Distribution of changes in the serum chloride and sodium concentrations under Diamox treatment in 23 refractory heart failure patients

		Change in serum sodium (mEq/L)	
		≥0	0>
Change in serum chloride (mEq/L)	≥0	14	6
	0>	2	2

Number of samples = 24; Male 17; Female 6; Age 57 ± 13 years (29–73); Daily dose of Diamox 250 mg–3 g/day; observation period 6.0 ± 2.8 days (3–12 days). Data obtained in the study by Relman *et al.*^7^

The specific diuretic action of this agent greatly contributes to favourably modulate serum electrolyte disturbances in HF pathophysiology. It should be kept in mind, however, that the efficiency of this diuretic agent to correct hyponatraemia would be modest, so worsening hyponatraemia even with the use of this agent should be carefully evaluated. Fortunately, this effect usually appears promptly, so the strategy can be quickly revised according to the results of blood test monitoring during a short observational period.

Both vasopressin receptor blockade^3^ and acetazolamide^9^ have similar effects to enhance the serum chloride concentration via their diuretic actions. Hence, according to my ‘chloride theory’ for HF pathophysiology,^6^ both diuretics could be expected to have a similar haemodynamic effect of redistributing the body fluid after diuresis, such as increasing or maintaining the plasma volume, and probably fluid drainage from the interstitial space due to changes in the serum osmolality/tonicity, mainly by serum chloride.^3^^,^^6^ While maintaining plasma volume might be beneficial for preserving the blood supply to the kidney and other organs, its persistent burden to the failing heart should be kept in mind^3^^,^^6^ when monitoring HF patients based on changes in BNP levels and haemodynamic parameters.

## Conclusion

It remains to be determined whether or not acetazolamide could be an alternative diuretic to vasopressin antagonists for some HF patients with hyponatraemia; compared with an oral vasopressin antagonist, acetazolamide is less costly and easy to administer both at outpatient clinic and hospitals. A recent pilot study^15^ has demonstrated that the addition of acetazolamide to the background regimen in patients with chronic HF exacerbations had additional diuretic effects and alleviated dyspnoea. Large randomized studies are required to re-evaluate the efficiency of this forgotten but useful agent for the treatment of HF and its potential for correcting hyponatraemia.

**Consent:** The author/s confirm that written consent for submission and publication of this case report including image(s) and associated text has been obtained from the patient in line with COPE guidance.

**Conflict of interest:** none declared.
